# Distribution and spread of tigecycline resistance gene *tet*(X4) in *Escherichia coli* from different sources

**DOI:** 10.3389/fcimb.2024.1399732

**Published:** 2024-06-28

**Authors:** Xin-Yan Fan, Yue Jiang, Han Wu, Jie Liu, Qing-Yun Gu, Zhen-Yu Wang, Lin Sun, Xinan Jiao, Qiuchun Li, Jing Wang

**Affiliations:** ^1^ Jiangsu Key Laboratory of Zoonosis/Jiangsu Co-Innovation Center for Prevention and Control of Important Animal Infectious Diseases and Zoonoses, Yangzhou University, Yangzhou, China; ^2^ Key Laboratory of Prevention and Control of Biological Hazard Factors (Animal Origin) for Agrifood Safety and Quality, Ministry of Agriculture of China, Yangzhou University, Yangzhou, China

**Keywords:** *tet*(X4), tigecycline resistance, public health, plasmid, mobile element

## Abstract

Tigecycline serves as a last-resort antimicrobial agent against severe infections caused by multidrug-resistant bacteria. Tet(X) and its numerous variants encoding flavin-dependent monooxygenase can confer resistance to tigecycline, with *tet*(X4) being the most prevalent variant. This study aims to investigate the prevalence and characterize tigecycline resistance gene *tet*(X) in *E. coli* isolates from various origins in Yangzhou, China, to provide insights into *tet*(X) dissemination in this region. In 2022, we tested the presence of *tet*(X) in 618 *E. coli* isolates collected from diverse sources, including patients, pig-related samples, chicken-related samples, and vegetables in Yangzhou, China. The antimicrobial susceptibility of *tet*(X)-positive *E. coli* isolates was conducted using the agar dilution method or the broth microdilution method. Whole genome sequencing was performed on *tet*(X)-positive strains using Illumina and Oxford Nanopore platforms. Four isolates from pig or pork samples carried *tet*(X4) and exhibited resistance to multiple antimicrobial agents, including tigecycline. They were classified as ST542, ST10, ST761, and ST48, respectively. The *tet*(X4) gene was located on IncFIA8-IncHI1/ST17 (n=2), IncFIA18-IncFIB(K)-IncX1 (n=1), and IncX1 (n=1) plasmids, respectively. These *tet*(X4)-carrying plasmids exhibited high similarity to other *tet*(X4)-bearing plasmids with the same incompatible types found in diverse sources in China. They shared related genetic environments of *tet*(X4) associated with IS*CR2*, as observed in the first identified *tet*(X4)-bearing plasmid p47EC. In conclusion, although a low prevalence (0.65%) of *tet*(X) in *E. coli* strains was observed in this study, the horizontal transfer of *tet*(X4) among *E. coli* isolates mediated by pandemic plasmids and the mobile element IS*CR2* raises great concerns. Thus, heightened surveillance and immediate action are imperative to curb this clinically significant resistance gene and preserve the efficacy of tigecycline.

## Introduction

Globally, the rapid increase of multidrug-resistant (MDR) bacteria poses a serious threat to clinical therapy and public health, a concern underscored by the World Health Organization (WHO, 2014). Tigecycline, a semisynthetic glycylcycline approved by the US Food and Drug Administration (FDA) in 2005, is considered as a last-resort antimicrobial agent for treating severe infections caused by MDR bacteria, particularly carbapenem-resistant Enterobacteriaceae (Pournaras et al., 2016; Yaghoubi et al., 2022). It acts by binding reversibly to the 30S ribosomal subunit, impeding the entry of tRNA into the A site of the ribosome and thereby preventing the elongation of peptide chains (Pournaras et al., 2016; Yaghoubi et al., 2022; Stein and Babinchak, 2012). In contrast to first and second generation tetracyclines (e.g., tetracycline, doxycycline, and minocycline), tigecycline can evade classical resistance mechanisms of tetracyclines, such as tetracyclines efflux pump Tet(A) and the ribosomal protection protein Tet(M) (Pournaras et al., 2016; Yaghoubi et al., 2022). However, the clinical use of tigecycline has led to the gradual emergence of strains resistant to tigecycline (Deng et al., 2014; Pournaras et al., 2016; Yaghoubi et al., 2022).

Overexpression of efflux pumps (AcrAB-TolC and OqxAB), mutations in the plasmid-mediated tetracycline efflux pump gene *tet*(A), and chromosomally located monooxygenase gene *tet*(X) and its variant *tet*(X2) can result in low-level tigecycline resistance (Moore et al., 2005; Yaghoubi et al., 2022). However, the recent discovery of novel plasmid-borne tigecycline resistance genes conferring high-level resistance, namely *tet*(X3) in *Acinetobacter baumannii* and *tet*(X4) in *Escherichia coli* from animals in China in 2019, has significantly compromised the clinical efficacy of tigecycline (He et al., 2019). Following the identification of *tet*(X3) and *tet*(X4), a variety of *tet*(X) variants associated with tigecycline resistance have been successively unveiled, with *tet*(X4) emerging as the predominant variant (Fang et al., 2020; Rueda Furlan et al., 2023). The mobile *tet*(X4) gene has increasingly been detected in *E. coli* from various sources, including animals, food products, humans, and the environment, primarily in China (He et al., 2019; Fang et al., 2020; Li et al., 2020b, 2023; Dong et al., 2022; Chen et al., 2019b; Li et al., 2020a). Its sporadic presence has also been reported in countries beyond China, such as Thailand, Pakistan, Vietnam, Singapore, the United Kingdom, and Norway (Ding et al., 2020; Li et al., 2022, 2023; Marathe et al., 2021; Martelli et al., 2022);. Previous research has indicated that mobile element IS*CR2* and various conjugative and mobilizable plasmids (e.g., IncQ, IncX1, IncFIB, and IncHI1) facilitate the horizontal transfer of *tet*(X4) in *E. coli* (He et al., 2019; Fang et al., 2020; Liu et al., 2022). IS*CR2* belongs to the IS*CR* elements, which can transpose adjacent DNA sequences via rolling-circle (RC) transposition, mediated by a single copy of the element (Toleman et al., 2006). IS*CR2* has been identified as a key factor in the dissemination of *tet*(X4) between plasmids and chromosomes across various species and hosts, forming the conserved transposable element IS*CR2*-*tet*(X4)-*catD* (Liu et al., 2022).

Although tigecycline is not approved for use in animals, the emergence of *tet*(X) and tigecycline resistance in farmed animals may be linked to the long-term and extensive use of tetracycline drugs, such as oxytetracycline and doxycycline in veterinary medicine (He et al., 2019). The rise and prevalence of the horizontally transferable tigecycline resistance gene *tet*(X4) in *E. coli* derived from animals and food sources raises concerns, due to the limited therapeutic options available. Consequently, there is an urgent need to assess and monitor the prevalence of *tet*(X) in pathogens. This study aims to investigate the prevalence and characterize *tet*(X4) in *E. coli* isolates from various origins in Yangzhou, China, to provide insights into the dissemination of *tet*(X4) in this region.

## Materials and methods

### Isolates and *tet*(X) detection

In 2022, a total of 618 *E. coli* isolates were collected from various sources, including patients (n=16), chicken meat (n=98), pork (n=108), chicken intestinal contents (n=86), pigs (n=241, feces and the pig farm environment), and vegetables (n=69) in Yangzhou, China (**Supplementary Table S1**). The samples were incubated in Luria-Bertani (LB) broth at 37°C for 18–24 h and then cultured on MacConkey agar at 37°C for 24 h. One pink colony per plate was further streaked on an eosin methylene blue (EMB) agar plate for 24 h at 37°C. One suspected *E. coli* isolate (metallic sheen color) was selected from each plate and subsequently confirmed by using matrix-assisted laser desorption/ionization time-of-flight mass spectrometry. The presence of the *tet*(X) gene was detected through PCR and Sanger sequencing using the universal primers *tet*(X)-F (5′-CCGTTGGACTGACTATGGC-3′) and *tet*(X)-R (5′-TCAACTTGCGTGTCGGTAA-3′) (Wang et al., 2019), with anticipated product of 475 bp. The *tet*(X) gene was amplified by PCR as follows: 94°C for 5 min; 30 cycles of penetration at 94°C for 30 s, annealing at 46°C for 30 s, and extension at 72 °for 30 s; and a 5 min final incubation at 72°C.

### Antimicrobial susceptibility testing

We assessed the susceptibility of *tet*(X)-positive *E. coli* isolates to 14 antimicrobial agents, which included ampicillin, cefotaxime, meropenem, gentamicin, amikacin, streptomycin, tetracycline, tigecycline, chloramphenicol, florfenicol, nalidixic acid, ciprofloxacin, colistin and sulfamethoazole/trimethoprim. Antimicrobial susceptibility testing was conducted using the agar dilution method or the broth microdilution method (limited to tigecycline and colistin). *E. coli* ATCC 25922 served as the quality control strain. Results were interpreted following the guidelines of the Clinical and Laboratory Standards Institute (CLSI) M100, 32^nd^ edition (CLSI, 2022). For tigecycline (≥ 1 mg/L), streptomycin (> 16 mg/L), and florfenicol (> 16 mg/L), interpretation was based on clinical breakpoint or epidemiological cut-off values established by the European Committee on Antimicrobial Susceptibility Testing (EUCAST; www.eucast.org).

### Conjugation experiments

Conjugation experiments were conducted using *tet*(X)-positive *E. coli* strains as donor strains and *E. coli* C600 (with high-level streptomycin resistance) or J53 (sodium azide-resistant) as the recipient strain. In brief, donor and the recipient strains were inoculated in 2 mL of LB broth at 37°C, 180 r/min for 4 h, followed by mixing in a 1:4 ratio (v/v) and further incubation at 37°C for 24 h. Transconjugants were selected on MacConkey agar plates contained tigecycline (2 μg/mL) and streptomycin (3000 μg/mL) or sodium azide (100 μg/mL) and subsequently confirmed by detecting *tet*(X) through PCR as described above. Experiments were performed in triplicate.

### Whole genome sequencing and analysis

The bacterial DNA from four *tet*(X4)-positive *E. coli* strains was extracted using the TIANamp Bacteria DNA Kit (Tiangen, Beijing, China). Subsequently, whole-genome sequencing was conducted using Illunina (NovaSeq) and Oxford Nanopore (MinION system) platforms. *De novo* hybrid assembly was carried out using Unicycler version 0.5.0 (https://github.com/rrwick/Unicycler) and further corrected using Pilon version 1.24. Genome sequences were analyzed for multilocus sequence typing (MLST), resistance genes, mutations, and plasmid replicons using the Centre for Genomic Epidemiology (CGE) pipeline (http://www.genomicepidemiology.org). Comparison of the *tet*(X4)-carrying plasmids with similar plasmids was conducted using BRIG (Alikhan et al., 2011).

### Nucleotide sequence accession number

The whole-genome sequences of the four *tet*(X4)-positive isolates have been deposited in GenBank under accession numbers: PRJNA1039986.

## Results

### Characterization of *tet*(X4)-positive *E. coli* isolates

In the present study, we observed a low prevalence (0.65%) of *tet*(X) in *E. coli* strains obtained from diverse sources. Two strains from pigs in the same pig farm and two strains from pork obtained from the same farm′s market were positive for *tet*(X4), respectively (**Table 1**). All *tet*(X4)-positive isolates displayed resistance to tigecycline (MIC ≥ 8 mg/L) and also exhibited resistance to ampicillin (n=4), gentamicin (n=1), streptomycin (n=3), tetracycline (n=4), chloramphenicol (n=4), florfenicol (n=4), nalidixic acid (n=1), and sulfamethoxazole/trimethoprim (n=4) (**Table 1**). However, they remained susceptible to cefotaxime, meropenem, amikacin, colistin, ciprofloxacin, and fosfomycin (**Table 1**). In addition to *tet*(X4), these isolates also harbored other resistance genes, such as *bla*_TEM-1b_ (n=4), *aadA22* (n=2), *strAB* (n=1), *tet*(A) (n=3), *qnrS1* (n=4), *floR* (n=4), *lnu*(G) (n=2), and *sul3* (n=3) (**Table 1**). In addition, strain YZ22MPE54 had a single mutation in *gyrA* (S83L), which was responsible for its resistance to nalidixic acid. The presence of these resistance genes and mutation likely accounts for their multidrug resistance profiles.

**Table 1:** Characterization of tigecycline-resistant *E. coli* strains in this study.

Strain	Sources	Sampling time	MLST	Resistance genes	Tigecycline MIC (mg/L)	Resistance patterns*^a^*	QRDR Mutation*^b^*	Location of *tet*(X4)
YZ22MPE6	Pork	2022.03.07	542	*bla*_TEM-1B_/*aadA22*/*tet*(X4)/*tet*(A)/*qnrS1*/*floR*/*lnu*(G)/*sul3*	8	AMP/STR/TET/TIL/CHL/FFC/SXT	N	IncFIA8-IncHI1/ST17
YZ22MPE54	Pork	2022.05.30	10	*bla*_TEM-1B_/*aadA22*/*tet*(X4)/*tet*(B)/*qnrS1*/*floR*/*lnu*(G)	16	AMP/STR/TET/TIL/CHL/FFC/NAL/SXT	*gyrA* (S83L)	IncFIA8-IncHI1/ST17
YZ22PE165	Pig feces	2022.06.02	761	*bla*_TEM-1B_/*tet*(X4)/*tet*(A)/*tet*(M)/*qnrS1*/*floR*/*sul3*/*dfrA5*/*mef*(B)	8	AMP/TET/TIL/CHL/FFC/SXT	N	IncFIA18-IncFIB(K)-IncX1
YZ22PE244	Pig feces	2022.06.02	48	*bla*_TEM-1A_/*aadA1*/*aadA2*/*aac(3)-IId*/ *strAB*/*tet*(X4)/*tet*(A)/*qnrS1*/*cmlA1*/*floR*/*sul2*/*sul3*/*dfrA12*/*lnu*(F)	16	AMP/GEN/STR/TET/TIL/CHL/FFC/SXT	N	IncX1

*^a^* AMP, ampicillin; GEN, gentamicin; STR, streptomycin; TET, tetracycline; TIL, tigecycline; CHL, chloramphenicol; FFC, florfenicol; NAL, nalidixic acid; SXT, ulfamethoxazole/trimethoprim.

*^b^* QRDR, quinolone resistance determining region, N, not found.

Based on whole-genome sequencing, the MLST analysis revealed that four *tet*(X4)-positive *E. coli* strains belonged to sequence types ST542, ST10, ST761, and ST48, respectively (**Table 1**). The *tet*(X4) gene was identified on three different types of plasmids: IncFIA8-IncHI1/ST17 (n=2), IncFIA18-IncFIB(K)-IncX1 (n=1), and IncX1 (n=1) (**Table 1**). However, all tigecycline-resistant isolates failed to transfer *tet*(X4)-carrying plasmids to *E. coli* C600 and *E. coli* J53 through conjugation.

### Characterization of the *tet*(X4)-carrying IncFIA8-IncHI1/ST17 plasmids pYUYZMPE6–1 and pYUYZMPE54 in *E. coli* strains YZ22MPE6 and YZ22MPE54

**Figure 1:**
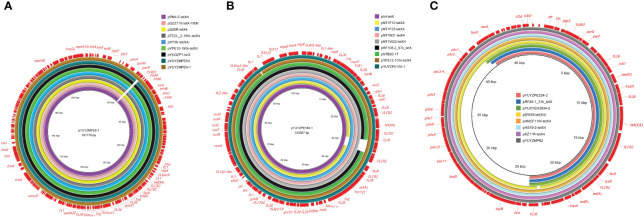
Sequence comparison of *tet*(X4)-carrying plasmids in this study with other similar plasmids using BRIG. **(A)** IncFIA8-IncHI1/ST17 plasmids pYUYZMPE6-1 and pYUYZMPE54 from *E coli* isolate YZ22MPE6 and YZ22MPE54; **(B)** IncFIA18-IncFIB(K)-IncX1 plasmid pYUYZPE165-1 from *E. coli* isolate YZ22PE165; **(C)** IncX1 plasmid pYUYZPE244-2 from *E. coli* isolate YZ22PE244. The outer circles in red with annotation are the reference plasmids pYUYZMPE6-1, pYUYZPE165-1, and pHS19-2-tetX4 (GenBank accession number MW940619), respectively.

The complete genome sequences of *E. coli* strains YZ22MPE6 (ST542) and YZ22MPE54 (ST10) were obtained, each consisting of one chromosome (4,607,660 or 4,670,021 bp) and one to four plasmids (**Supplementary Table S2**). The *tet*(X4) gene in isolates YZ22MPE6 and YZ22MPE54 was located on IncFIA8-IncHI1/ST17 plasmids pYUYZMPE6–1 (191,776 bp) and pYUYZMPE54 (190,712 bp), respectively. Both plasmids carried five additional resistance genes including *bla*_TEM-1b_, *aadA22*, *floR*, *tet*(A), and *qnrS1*. They were almost identical to each other, except for the insertion of one copy of insertion sequence IS*903* downstream of the *parR* gene in pYUYZMPE6–1, with 9-bp direct repeats (DR, 5’-CTCCAGTTA-3’). They also displayed high similarity (>99.9%) to other *tet*(X4)-carrying IncFIA8-IncHI1/ST17 plasmids isolated from various Enterobacteriaceae species in China, for instance, pPM4–5-tetX4 (*E. coli*, pig cecum, CP096833), pQZZ116-tetX-190K (*E. fergusonii*, pig feces, CP095844), pSZ6R-tetX4 (*Citrobacter* sp., pork, MW940627), pTECL_2–190k-tetX4 (*Enterobacter cloacae*, swine nasal swab, MZ773210), pXY36-tet(X4) (*Morganella morganii*, swine, ON390820), and pYPE10–190k-tetX4 (*E. coli*, pork, CP041449), and pYUGZP1-tetX (*E. coli*, pig, MW439255) (**Figure 1A**).

### Characterization of the *tet*(X4)-carrying IncFIA18-IncFIB(K)-IncX1 plasmid pYUYZPE165–1 in *E. coli* strain YZ22PE165

The complete genomic sequences of strain YZ22PE165 was obtained, comprising one chromosome (4,711,790 bp) and three plasmids named pYUYZPE165–1 to pYUYZPE165–3 (**Supplementary Table S2**). Notably, the largest plasmid, designated as pYUYZPE165–1, harbored *tet*(X4) and eight additional resistance genes [*bla*_TEM-1b_/*tet*(A)/*tet*(M)/*qnrS1*/*floR*/*sul3*/*dfrA5/mef*(B)]. This plasmid belonged to the hybrid IncFIA18-IncFIB(K)-IncX1 plasmid with a size of 103,087 bp. *E. coli* ST761 has been identified as a successful clone for transferring *tet*(X) associated with IncFIA18-IncFIB(K)-IncX1 plasmid across different sources in China (Li et al., 2020a, b; Li et al., 2021b; Wang et al., 2022; Zhai et al, 2022b). As shown in **Figure 1B**, pYUYZPE165–1 exhibited high similarity to *tet*(X4)-carrying IncFIA18-IncFIB(K)-IncX1 plasmids found in ST761 *E. coli* strains in China, with identities ranging from 99.88% to 99.98% and coverage from 96.17% too 100.00%, such as plasmids pNT1F10-tetX4 (CP075463), pNT1W22-tetX4 (CP075470), pRF108–2_97k_tetX (MT219820), and pSTB20–1T (CP050174) from pigs, p54-tetX (cow, CP041286) from a cow, and pYPE12–101k-tetX4 (CP041443) from a pork sample. Similar *tet*(X4)-carrying IncFIA18-IncFIB(K)-IncX1 plasmids have also been reported in other *E. coli* lineages, such as pNT1N31-tetX4 (pig, CP075481) and pNT1F25-tetX4 (pig, CP075471) obtained from ST716 and ST1421, respectively (Li et al., 2021b) (**Figure 1B**).

### Characterization of the *tet*(X4)-carrying IncX1 plasmid pYUYZPE244–2 in *E. coli* strain YZ22PE244

The complete sequence of isolate YZ22PE244 was obtained. It consisted of a single chromosome (4,515,595 bp) and four plasmids, as detailed in **Supplementary Table S2**. Among them, *tet*(X4) and three additional resistance genes [*aadA2*, *tet*(A), *lnu*(F)] were found co-located on a 30,871 bp IncX1 plasmid designated as pYUYZPE244–2. This *tet*(X4)-carrying plasmid was similar to other IncX1 plasmids carrying *tet*(X4), such as pYUYZMP62 (MW439254, pig), pDW28-tet(X4) (ON390803, pig), pYY76–1-2 (CP040929, cow), pRF45–1_31k_tetX (MT219821, pig) and pYUXYEH3934–2 (CP110980, patient) from *E. coli* in China, pHNCF11W-tetX4 (CP053047, chicken) and pSZ11R-tetX4 (MW940629, pork) from *Escherichia*, and pHS19–2-tetX4 (MW940619, pork) from *Klebsiella* sp. (**Figure 1C**). However, pYUYZPE244–2 as well as plasmids pDW28-tet(X4), pRF45–1_31k_tetX and pYUXYEH3934–2 lacked the conjugative transfer region of IncX1 plasmids (**Figure 1C**), potentially explaining their failure in conjugation (Sun et al., 2023).

### Related genetic environments of *tet*(X4) in four plasmids

**Figure 2:**
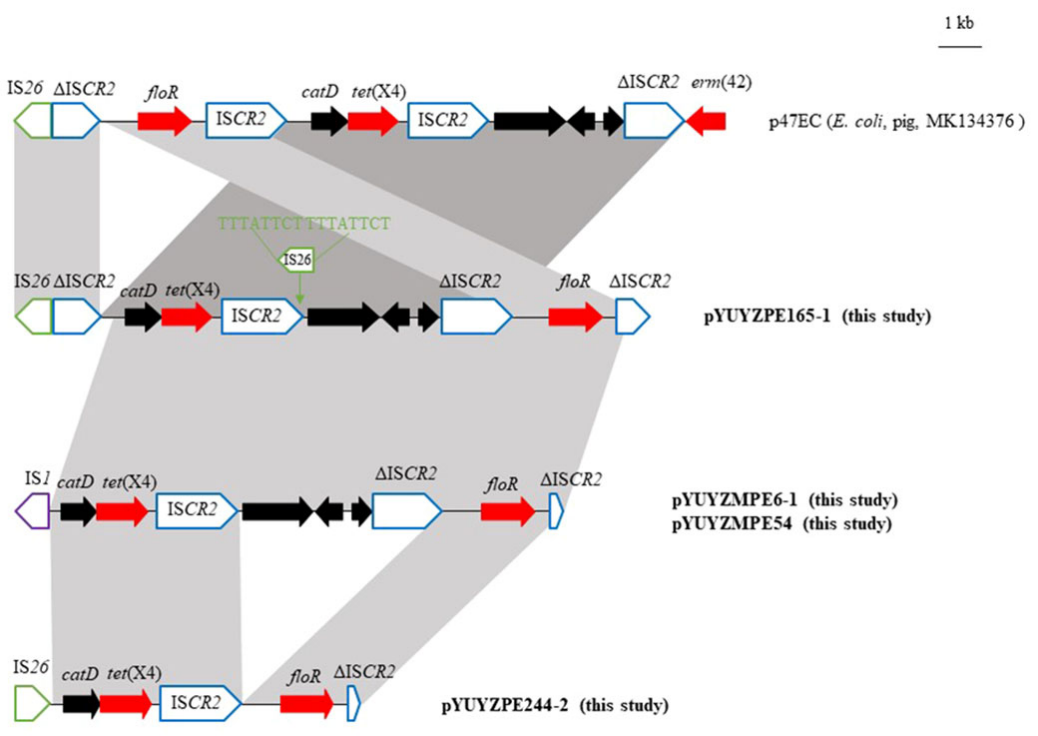
Genetic organization of the *tet*(X4) gene in this study and comparison with p47EC (MK134376). The extents and direction of antibiotic resistance (red arrows) and other genes (black arrows) are indicated. ISs are shown as boxes labeled with their name. labeled vertical arrows with IS box indicate the insertion site of IS element. Δ indicates a truncated mobile element. Direct repeats are indicated by arrows and sequences.

Four *tet*(X4)-carrying plasmids in this study shared related genetic environments of *tet*(X4) to the first identified *tet*(X4)-bearing plasmid p47EC (170,312 bp, IncFIB) from *E. coli* strain 47EC, isolated from a fecal swab from a pig farm in China in 2018 (He et al., 2019). The *tet*(X4) gene was situated within a 6,456-bp region IS*CR2*-*catD*-*tet*(X4)-IS*CR2* in p47EC, capable of forming a classical rolling circle transposable unit (RC-TU) mediated by IS*CR2* (He et al., 2019). However, among the four *tet*(X4)-carrying plasmids in this study, intact IS*CR2* was found only downstream of *tet*(X4), although one copy of IS*26* was inserted into the origin of replication (*ori*IS) of IS*CR2*, flanked with 8-bp DRs (5’-TTTATTCT-3’) in pYUYZPE165–1(**Figure 2**). Upstream of *catD*-*tet*(X), the sequences were truncated by other insertion sequences, namely IS*26* (pYUYZPE244–2 and pYUYZPE165–1) and IS*1* (pYUYZMPE54 and pYUYZMPE6–1), resulting in a truncated IS*CR2* upstream in pYUYZPE165–1 and the absence of IS*CR2* in the remaining three plasmids (**Figure 2**). In contrast to p47EC, our four plasmids contained the florfenicol resistance gene *floR* downstream of the *tet*(X4) module, associated with an incomplete IS*CR2* (**Figure 2**).

## Discussion

Since its discovery in *E. coli* in 2019, the plasmid-mediated tigecycline resistance gene *tet*(X4) has been identified in various bacterial species, such as *E. coli*, *Proteus*, *Raoultella ornithinolytica*, *Acinetobacter baumannii*, *Aeromonas caviae*, *Citrobacter freundii*, *Klebsiella pneumoniae*, and *Shewanella xiamenensis*, with *E. coli* being the primary host (He et al., 2019; Dong et al., 2022; Chen et al., 2019a; Dao et al., 2022; Zeng et al., 2021; Zhai et al., 2022a). In our study, we tested *tet*(X4) in *E. coli* from diverse sources, revealing the exclusive presence of *tet*(X4) in *E. coli* originating from pigs and pork. Conversely, *tet*(X4) was not detected in *E. coli* isolates derived from other sources, including chickens, chicken meat, vegetables, and clinical specimens. However, it is imperative to acknowledge that the number of isolates from these sources was relatively limited, posing a constraint on our study. Currently, *tet*(X4) is commonly detected in *E. coli* originating from food-producing animals and food products, particularly pigs and pork, but it is infrequently reported in *E. coli* from humans (He et al., 2019; Sun et al., 2019a, 2020; Dong et al., 2022; Li et al., 2021a; Sun et al., 2023). The detection rate of *tet*(X4) in this study was relatively low (0.65%), aligning closely with the findings of Sun et al. (0.22%) (Sun et al., 2019a), Yu et al. (0.17%) (Yu et al., 2022), and Zhang et al. (0.32%) (Zhang et al., 2020) in their research on *E. coli* collected from China. However, in contrast, significantly higher detection rates of *tet*(X4) were reported in retail pork samples from Shandong and Sichuan provinces (20.6%, 7/34) (Bai et al., 2019) and across ten regions in China (39.56%, 55/139) (Li et al., 2021a).

Currently, a variety of *E. coli* strains with different sequence types (STs) carrying *tet*(X4) have been identified in animals, food, the environment, and humans (Li et al., 2020b, 2023; Sun et al., 2019b, 2020; Dong et al., 2022; Li et al., 2021a, b; Cui et al., 2022). Additionally, *tet*(X4)-positive plasmids with the same Inc type have been detected in *E. coli* strains sharing the same or different STs (Sun et al., 2019b, 2020; Li et al., 2021a; Cui et al., 2022). These observations suggest that plasmids are the primary means by which *tet*(X4) is disseminated among diverse *E. coli* strains. However, it is worth noting that the clonal spread of certain ST-type *E. coli* strains may also contribute to the dissemination of *tet*(X4). For instance, *E. coli* ST542, ST10, ST48, and ST761, which were identified in our study, represent common ST types known to carry *tet*(X4) in China and have been detected in animals, food products, the environment, and patients (Dong et al., 2022; Bai et al., 2019; Chen et al., 2019c; Zhang et al., 2020; Li et al., 2021a; Cui et al., 2022; Wang et al., 2022; Zhai et al., 2022b). Globally, ST10 and ST48 are the most prevalent clones in the *tet*(X)-positive *E. coli* isolates, detected in multiple countries and over 10 hosts (Li et al., 2023). Notably, certain *tet*(X)-positive *E. coli* isolates (e.g., ST10) from different geographical locations or hosts shared high genetic similarity (<200 SNPs), indicating possible clonal transmission (Li et al., 2023).

Plasmids are crucial vectors for *tet*(X4) transmission. Presently, *tet*(X4) is associated with diverse plasmids, such as IncX1, IncFIA/IncHI1, IncFIA18/IncFIB(K)/IncX1, IncFII, IncQ1, ColE2, IncA/C, and IncN plasmids (Fang et al., 2020; Li et al., 2020b; Li et al., 2021b; Cui et al., 2022; Wang et al., 2022). The *tet*(X4)-bearing plasmids identified in this study exhibit high similarity to those *tet*(X4)-carrying plasmids found in Enterobacteriaceae, particularly *E. coli*, of various origins. This observation underscores the role of horizontal transmission of prevalent plasmids in the dissemination of *tet*(X4) within Enterobacteriaceae. Certain plasmids with a complete conjugal transfer region carrying *tet*(X4) possess conjugative capabilities, allowing them to be horizontally transferred between distinct bacterial strains, greatly facilitating the widespread distribution of *tet*(X4). Conversely, plasmids lacking a conjugal transfer region, such as the *tet*(X4)-positive IncFIA18-IncFIB(K)-IncX1 plasmids in this study, are rendered non-conjugative. For instance, the IncX1 plasmid has been identified in *E. coli* from various sources and has demonstrated its capacity to transfer and persist within a range of Enterobacteriaceae (Li et al., 2020b; Cui et al., 2022; Chen et al., 2019c). However, our study found that the IncX1 plasmid pYUYZPE244–2 was incapable of conjugative transfer, possibly due to the absence of the conjugal transfer region compared to other *tet*(X4)-positive IncX1 plasmids. On the contrary, the presence of IncI2, IncX4, and IncFII conjugative plasmids carrying *mcr-1* could serve as helper plasmids, enabling the conjugative transfer of non-conjugative *tet*(X4)-positive IncX1 or IncQ plasmids (Sun et al., 2019b; He et al., 2020). Furthermore, the non-conjugative *tet*(X4) plasmid can undergo homologous recombination with conjugative IncFII plasmid mediated by IS*26*, resulting in the formation of a novel fusion plasmid, thus, achieving the conjugative transfer of *tet*(X4) (Du et al., 2020). In our investigation, we also discovered that plasmids carrying *tet*(X4) often bear a variety of drug resistance genes, such as *bla*_TEM-1b_, *qnrS1*, and *floR*, which confer resistance to β-lactams, quinolones, and florfenicol. It suggests that these commonly used antibiotics in livestock may exert a co-selective pressure on *tet*(X4).

The horizontal transfer of *tet*(X4) between different plasmids, plasmids or chromosomes is associated with the insertion sequence IS*CR2* (He et al., 2019; Chen et al., 2019b; Liu et al., 2022). IS*CR2* is present upstream and/or downstream of *tet*(X4) and other *tet*(X) variants such as *tet*(X3), *tet*(X5), and *tet*(X6) (He et al., 2019; Wang et al., 2019; He et al., 2020; Liu et al., 2022). As revealed in prior research, IS*CR2* carries adjacent regions including *tet*(X4) to form a 4,608 bp RC-TU, and it could also capture more resistance genes such as *floR* to form a larger RC-TU, although the IS*CR2*-*tet*(X4) element was frequently truncated by other insertion sequences such as IS*26* (Liu et al., 2022). Additionally, the insertion sequence IS*1* can also mediate the transfer of *tet*(X4) (Yu et al., 2022).

## Conclusions

While the prevalence of *tet*(X4) within *E. coli* isolates in this study was relatively low, the horizontal transfer of this resistance gene within *E. coli* strains associated with pandemic plasmids (IncFIA8-IncHI1, IncFIA18-IncFIB(K)-IncX1, and IncX1) and mobile elements such as IS*CR2* is a matter of great concern. Mobile elements and plasmids play a pivotal role in rapid dissemination of this clinically crucial resistance gene in *E. coli* originating from livestock and animal-derived food products. It has the potential for this gene to be transmitted to humans through the food chain. The detection of *tet*(X4) among *E. coli* isolates from animals and food products raises substantial public health concerns, necessitating enhanced surveillance and immediate action to control this medically significant resistance gene and uphold the efficacy of tigecycline.

## Data availability statement

The datasets presented in this study can be found in online repositories. The names of the repository/repositories and accession number(s) can be found in the article/**Supplementary Material**.

## Ethics statement

The studies involving humans were approved by Jiangsu Key Laboratory of Zoonosis, Yangzhou University. The studies were conducted in accordance with the local legislation and institutional requirements. The human samples used in this study were acquired from gifted from another research group. Written informed consent for participation was not required from the participants or the participants’ legal guardians/next of kin in accordance with the national legislation and institutional requirements. The animal studies were approved by Jiangsu Key Laboratory of Zoonosis, Yangzhou University. The studies were conducted in accordance with the local legislation and institutional requirements. Written informed consent was obtained from the owners for the participation of their animals in this study.

## Author contributions

X-YF: Investigation, Writing – original draft. Y-J: Formal analysis, Investigation, Writing – review & editing. HW: Writing – original draft. JL: Investigation, Writing – review & editing. Q-YG: Investigation, Writing – review & editing. Z-YW: Formal analysis, Software, Writing – review & editing. LS: Conceptualization, Resources, Writing – review & editing. XJ: Funding acquisition, Writing – review & editing. QL: Conceptualization, Writing – review & editing. JW: Conceptualization, Formal analysis, Supervision, Writing – original draft, Writing – review & editing.
