# Supplementary material for: Distribution and spread of tigecycline resistance gene tet(X4) in Escherichia coli from different sources
Source: Front Cell Infect Microbiol. 2024 Jun 28;14:1399732. doi: 10.3389/fcimb.2024.1399732 (PMC11239352; doi:10.3389/fcimb.2024.1399732)
Supplement: Supplementary file 1 [file Table_1.docx]

**Table S1.** Information of *tet*(X)-carrying *E. coli* from different sources in Yangzhou area

| **Sources** | **Sampling time** | **Samples (no.)** | ***E. coli* (no.)** | ***tet*(X)-positive *E. coli* (no.)** |
| --- | --- | --- | --- | --- |
| patients | 2022.01-2022.08 | / | 16 | 0 |
| chicken meat | 2022.03-2022.05 | 118 | 98 | 0 |
| pork | 2022.03-2022.05 | 117 | 108 | 2 |
| chicken intestinal contents | 2022.06 | 159 | 86 | 0 |
| pig farms  (feces and the environment) | 2022.05-2022.06 | 299 | 241 | 2 |
| vegetables | 2022.07 | 248 | 69 | 0 |

**Table S2**. Complete genome sequences of *tet*(X4)-carrying *E. coli* strains in this study.

|  | **Size (bp)** | **Resistance genes** | **Plasmid replicon** |
| --- | --- | --- | --- |
| **YZ22MPE6** |  |  |  |
| chromosome | 4,607,660 | none |  |
| pYUYZMPE6-1 | 191,776 | *bla*_TEM-1B_, *aadA22*, *tet*(X4), *qnrS1*, *floR*, *lnu*(G) | IncFIA/IncHI1 |
| pYUYZMPE6-2 | 65,304 | none | IncY |
| pYUYZMPE6-3 | 32,331 | *bla*_TEM-1B_, *tet*(A), *qnrS1*, *sul3* | IncX1 |
| pYUYZMPE6-4 | 2,028 | none | NT |
| **YZ22MPE54** |  |  |  |
| chromosome | 4,670,021 | *tet*(B) |  |
| pYUYZMPE54 | 190,712 | *bla*_TEM-1B_, *aadA22*, *tet*(X4), *qnrS1*, *floR*, *lnu*(G) | IncFIA/IncHI1 |
| **YZ22PE165** |  |  |  |
| chromosome | 4,711,790 | none |  |
| pYUYZPE165-1 | 103,087 | *bla*_TEM-1B_, *tet*(A), *tet*(M), *tet*(X4), *qnrS1*, *floR*, *sul3*, *dfrA5*, *mef*(B) | IncFIA/IncFIB(K)/IncX1 |
| pYUYZPE165-2 | 4,145 | none | NT |
| pYUYZPE165-3 | 4,072 | none | NT |
| **YZ22PE244** |  |  |  |
| chromosome | 4,515,595 | none |  |
| pYUYZPE244-1 | 218,363 | *bla*_TEM-1A_, *aadA1*, *aadA2*, *aac(3)-IId*, *strAB*, *tet*(A), *qnrS1*, *cmlA1*, *floR*, *dfrA12*, *sul2*, *sul3* | IncFIB(K)/IncFIB |
| pYUYZPE244-2 | 30,871 | *aadA2*, *tet*(X4), *tet*(A), *lnu*(F) | IncX1 |
| pYUYZPE244-3 | 4,715 | none | NT |
| pYUYZPE244-4 | 4,664 | none | NT |

NT, not typeable.
